# RELATIONSHIPS, RIGHTS, AND RESPONSIBILITIES: (RE)VIEWING THE NHS CONSTITUTION FOR THE POST-PANDEMIC ‘NEW NORMAL’

**DOI:** 10.1093/medlaw/fwac028

**Published:** 2022-08-26

**Authors:** Caroline A B Redhead, Sara Fovargue, Lucy Frith, Anna Chiumento, Heather Draper, Paul B Baines

**Affiliations:** School of Law, Centre for Social Ethics and Policy, The University of Manchester, Manchester, UK; School of Law, The University of Sheffield, Sheffield, UK; School of Law, Centre for Social Ethics and Policy, The University of Manchester, Manchester, UK; Institute of Population Health, Department of Primary Care and Mental Health, University of Liverpool, Liverpool, UK; Warwick Medical School, University of Warwick, Coventry, UK; Warwick Medical School, University of Warwick, Coventry, UK

**Keywords:** COVID-19, ‘new normal’, NHS Constitution, relationality, responsibilities, rights

## Abstract

Action needs to be taken to map out the fairest way to meet the needs of all NHS stakeholders in the post-pandemic ‘new normal’. In this article, we review the NHS Constitution, looking at it from a relational perspective and suggesting that it offers a useful starting point for such a project, but that new ways of thinking are required to accommodate the significant changes the pandemic has made to the fabric of the NHS. These new ways of thinking should encompass concepts of solidarity, care, and (reciprocal) responsibility, grounded in an acceptance of the importance of relationships in society. To this end, we explore and emphasise the importance of our interconnections as NHS stakeholders and ‘re-view’ the NHS Constitution from a relational perspective, concentrating on the rights and responsibilities it describes for patients and the public as NHS stakeholders. We argue that the NHS Constitution, of which most stakeholders are probably unaware, can be used as a tool to engage us, and to catalyse conversation about *how* our responsibilities as NHS stakeholders should change in the post-pandemic ‘new normal’.

## I. INTRODUCTION

The coronavirus (COVID-19) pandemic dominated 2020 and 2021, and is likely to be a feature of our lives for some time to come. It has caused governments worldwide to rethink how health services operate, further exposed systemic inequity, and caused human suffering on an immense scale. The NHS has been central to the response to the pandemic in the UK. The extreme and distressing working conditions NHS staff have experienced, and continue to tolerate, have been widely publicised.^1^ Government public health messaging, urging the public to stay home, protect the NHS, and save lives, has emphasised the centrality of solidarity as a community response to the NHS effort,^2^ where solidarity is understood as an affirmation of others’ suffering and an expression of tangible support.^3^ Against this background, our purpose in this paper is 2-fold. First, we offer the practical suggestion that the NHS Constitution, currently not well used or understood,^4^ could usefully be re-purposed to support a conversation about how patients and the public can continue to support the NHS into the post-pandemic ‘new normal’^5^ and beyond, and equally as important, what it is reasonable to expect from the NHS as the pandemic gives way to endemic COVID-19. Secondly, we advocate a change of philosophical perspective to underpin our practical suggestion. We argue that the importance of relationships across society must be acknowledged, and our central project is to re-view the NHS Constitution through the lens of relationality.

The importance of relationships and communities has been underlined by different experiences of the pandemic across society. The virus has disproportionately affected the health of people with certain characteristics (eg ethnicity, sex, and age), and those unable to work from home have been at a greater risk of contracting SARS-C0V-2.^6^ Some of the hardships of the response to the pandemic, such as painful separation of family and friends, were shared across the community, but our capacity to protect ourselves from the virus itself was different.^7^ As the pandemic has developed, it has become obvious that some have found themselves in more perilous situations than others. If the NHS is to continue to offer a safe berth to all stakeholders, now is the time to consider *how* this might be achievable. As the NHS emerges from the grip of the pandemic, action is urgently required to map out the best way to attend to the health and welfare needs of all NHS stakeholders—public, patients, *and* staff in the ‘post-pandemic’ new normal.^8^ In the UK, with attention turning to how we live with COVID-19 as an endemic disease, we are moving into the ‘post-pandemic’ new normal. Regular vaccination is likely to become the norm, and re-infection with (a variant of) COVID-19 to be common, even with vaccination. The characteristics of this new normal are being shaped as we go, but questions of fairness and equity are likely to be defining characteristics.^9^ Questions of fairness and equity are big questions, which we cannot hope fully to address here. We will, however, in re-viewing the responsibilities of NHS stakeholders (in section V) consider in more detail the social determinants of health, a fairer distribution of health, and the importance of narrowing health inequalities. These questions, already critical following a decade of widening health inequalities,^10^ have been exacerbated by the effects of the virus on NHS waiting lists and operation backlogs, which sit at record levels,^11^ and have thrown important questions relating to prioritisation, communication, and engagement with patients into even sharper relief.^12^

The consequences of the pandemic in the post-pandemic ‘new normal’ will be multi-faceted and intergenerational. Decision-makers and policy-writers will, for some time, be developing and promulgating guidelines and frameworks without a full understanding of the events yet to unfold. While the UK has experienced suffering and death on a scale probably unprecedented in living memory, the pandemic has also energised innovation, collaboration and, at least in the early stages, values-based actions within communities, as people stood in solidarity with each other, and with NHS healthcare workers. Similar responses were seen within and between communities locally, nationally, and internationally.^13^ The demands made by the pandemic on NHS staff members, and the physical and emotional suffering they have endured as a direct consequence, visibly engaged the attention and support of the public (for a time at least).^14^ There has been a sense of social obligation to support the ability of the NHS to care for those with COVID-19 and to protect those needing urgent healthcare for other reasons. In supporting the NHS (even if not in all other respects), we have, as a national community, ‘all been in it together’,^15^ staying at home, protecting our NHS, and saving lives.^16^

This increased sense of community and social obligation has thrown into sharper relief a number of matters, not least the relative order of things in our society and, relatedly, the importance of ethics and values in shaping our society and how it operates. The vulnerabilities of disabled and older people and the care system in which they live have been particularly highlighted during the pandemic.^17^ As have the complex relationships between individual members of ‘the public’, the public as a collective, and our public institutions. Social justice, health equity, questions of safety, and tolerable levels of risk have become matters of general concern. Reporting its review of the effects of the pandemic on health inequalities in England, the 2020 Marmot Report recommended that urgent action is required to address health inequalities, in particular by paying attention to the social determinants of health. Building on the findings of their two previous reports,^18^ Marmot and colleagues reiterated their earlier conclusion that the lower a person’s social position, the higher the chances of experiencing poorer health outcomes, stressing that action on health inequalities, therefore, requires urgent action across *all* the social determinants of health.^19^ Further, the 2020 Report highlighted the link between inequalities in social and economic conditions before the pandemic and the high and unequal death toll from COVID-19. Arguing that high levels of inequality are incompatible with a fair and healthy society, Marmot and colleagues have emphasised the importance of reducing health inequalities in the post-pandemic new normal.^20^

Although responsibility for attending to, and reducing, health inequalities does not, and cannot, lie with the NHS in isolation,^21^ the NHS will have an important role to play. It is crucial that the values underpinning decision-making about access to services and are transparent, not least because many people, who have been asked to wait whilst COVID-19 patients have taken priority, may be asked to continue to wait if others’ needs are considered more urgent. Emphasising that the nation’s health should be the highest priority for government as we rebuild from the pandemic, Marmot and others have recommended that the reduction of health inequalities requires the implementation of long-term policies with equity at their heart.^22^

In this paper, we suggest re-viewing the NHS Constitution from a relational perspective offers an opportunity for the transparent and inclusive development of such equitable policies. By ‘relational’, we mean a perspective that acknowledges that each individual is in basic ways constituted by the networks of relationships of which they are a part.^23^ We start, in section II, by discussing the policy context from which the NHS emerged, and briefly consider its development, concentrating on the relational values which constitute it. We then introduce the NHS Constitution, highlighting the importance it places on the relational engagement of all stakeholders in the NHS. In section III, we explore relational ideas and approaches in more detail. These provide the context for our contention that relationships are crucial in starting to address the questions of health inequality that we suggest must be answered in the ‘post-pandemic’ new normal. In sections IV and V, we offer a new analysis of the rights and responsibilities at the heart of the NHS Constitution, situating this analysis in the historical and relational context described in sections II and III. We conclude, in section VI, by suggesting that the relational, values-based NHS Constitution provides a means of reinvigorating the solidarity we argue was demonstrated as an initial response to the pandemic, and carrying it forward.

## II. THE HISTORICAL CONTEXT: THE CONSTITUTING OF THE NHS AND THE NHS CONSTITUTION

### A. *The constituting of the NHS*

Aneurin Bevan, generally recognised as the founder of the NHS, saw its creation as a way to displace the predominantly market-based model of healthcare that prevailed prior to its creation, with one based on communal responsibility.^24^ His focus was a community whose ‘social codes [had] the collective well-being for their aim’.^25^ In the context of a health service, he was convinced that:

the collective principle asserts that the resources of medical skill and the apparatus of healing shall be placed at the disposal of the patient, without charge, when he or she needs them; that medical treatment and care should be a communal responsibility; that they should be made available to rich and poor alike in accordance with medical need and by no other criteria.^26^

Kenneth Veitch has suggested that this idea of ‘communal responsibility’ can be understood as a form of solidarity, in which the state’s obligation to provide health services to its citizens, free at the point of need, is embedded.^27^ Bevan was committed to social justice, and this was reflected in his desire to build a health service within which universal access to healthcare was guaranteed. He wanted to ensure that medical treatment and care were placed at the disposal of the patient, without charge, in response only to medical needs and without reference to an ability to pay.^28^ Bevan saw a link between financial anxiety and illness; specifically, that financial hardship was a ‘serious hindrance’ to recovery.^29^ He wanted, by facilitating universal access to the social good of healthcare, in accordance with need and without charge, to ameliorate these harms.

His critics contended that universal access to healthcare would give rise to a ‘something for nothing’ culture.^30^ Bevan disagreed (‘to call it something for nothing is absurd because everything has to be paid for in some way or another’),^31^ arguing that the selection of general taxation as the financing model for the NHS would, in fact, embed the central idea of communal responsibility underpinning his vision for the NHS. He considered that taxpayers, as stakeholders in the NHS, provided a service to others that would be available to them if and when they were to become ill.^32^ Veitch has suggested that this method of financing introduced principles similar to the Roman Law notion of *obligatio in solidum*, or a responsibility undertaken by each member of a group of people to the welfare of others in the group if and when they need help.^33^ He has thus argued that Bevan’s project was to ensure that obligation and solidary values were *constitutive* of the NHS as a healthcare system, grounding it in ideas of solidarity and communal responsibility, in active public participation in the co-production of a common good.^34^ We too emphasise the importance of solidarity, understood in the way that Veitch has described it. Characterising solidarity as encompassing a responsibility for the welfare of others in a particular group is (as we discuss in section III) underpinned by an acknowledgement of interdependence and mutuality. These notions, of solidarity, interdependence, and mutuality, assume the importance of *relationships* in a health service whose stakeholders are engaged in the production of a common good, and thus we are suggesting that notions of relationality have always been at the heart of the NHS that Bevan wanted to build.

### B. *The NHS Constitution*

Established by section 1 of the Health Act 2009, the first NHS Constitution, and a Handbook providing additional information about it, were published in January 2009.^35^ The NHS Constitution is essentially a framework, organised into four sections. The first and second sections explain the principles and values that underpin and guide the NHS. The third section is a guide to patients’ rights and the responsibilities that patients and the public have for looking after their own health, for working in partnership with NHS staff, and for using NHS resources well and sustainably. The fourth section explains the rights of NHS staff (as stakeholders in the NHS) and the expectations the NHS has of them. The Handbook is designed to give additional information about every aspect of the NHS Constitution, and is a resource for organisations that offer support and advice.^36^ The 2009 Act requires the Secretary of State for Health and Social Care to review and republish the NHS Constitution at least once every 10 years and to review and republish the Handbook to the NHS Constitution every 3 years.^37^ The Secretary of State is also required to report to Parliament on the effect of the NHS Constitution every 3 years.^38^

NHS bodies (and private and voluntary sector providers supplying NHS services) are required by the 2009 Act ‘to have regard to’ the NHS Constitution in their decisions and actions.^39^ This statutory duty, described as a ‘target duty’ (in that a duty to ‘have regard’ to something describes broad objectives rather than specifying a particular or precisely defined end result^40^), obliges decision-makers to take account of the NHS Constitution in their decision-making and to justify departing from the expectations it outlines.^41^ The intention behind the duty to ‘have regard’ to the NHS Constitution was that its values and principles be embedded at every level within the health service and among those organisations providing NHS services.^42^

The NHS Constitution was also intended to embed the principle of stakeholder involvement, and the engagement of patients, staff, and the public in developing it was considered key.^43^ The aim was to secure enduring meaning and value to stakeholders, and evoke a sense of shared ownership.^44^ For patients, for example, the NHS Constitution was supposed to provoke challenge and shared responsibility for making the best use of NHS services. For staff, the rights and responsibilities were intended to empower them to develop better services for patients and improve engagement with their employers.^45^ The NHS Constitution might, therefore, be described as representing an aspirational standard, more in the nature of a declaration, or a mission statement. It:

establishes the principles and values of the NHS in England. It sets out rights to which patients, public and staff are entitled, and pledges which the NHS is committed to achieve, together with responsibilities which the public, patients and staff owe to one another to ensure that the NHS operates fairly and effectively.^46^

Thus, the NHS Constitution explicitly reiterates Bevan’s thesis about the crucial importance of ‘the collective principle’ to the NHS,^47^ where the NHS is understood as a community, ‘whose social codes have collective well-being for their aim’.^48^ To build on this, it is to a consideration of *relationality* that we now turn to ground and shape our argument that the NHS Constitution is built on relational values, and that these should direct a re-view of the NHS Constitution for the post-pandemic new normal.

## III. RELATIONALITY AND THE NHS CONSTITUTION

The importance of public health infection prevention and transmission control measures during the pandemic has resulted in a ‘frame shifting’ in the NHS from an individual patient perspective to a population-based, public health perspective.^49^ This does not mean that respect for individual rights has necessarily become less prominent, rather that, as we will contend in sections IV and V, the ‘frame shifting’ has changed the context for *interpreting* the rights and responsibilities of patients and the public. The difference, we propose, is the increased importance of the *relationships* within which the rights and responsibilities of NHS stakeholders (patients, the public, and members of NHS staff) are experienced and enacted, and the values that inform them. This ‘frame shifting’ necessarily acknowledges and emphasises the importance of relational thinking.

### A. *A Relational Turn: Relational Thinking and the NHS Constitution*

Theorists of relational approaches understand individuals to be continually constituted by the relational processes in which they engage and are engaged.^50^ Jennifer Nedelsky, whose theories of relationality we explore in more detail in section IV, has considered relationality from the perspective of law. She argues for a language of law that moves away from an emphasis on limits and boundaries, and rejects the underlying concept of the ‘bounded’ self, in favour of an emphasis on the *relationships* that law fosters and reflects.^51^ This rejection of ‘boundaries’ is a feature of relational thinking more generally, as a means of transcending paradigms and theorising the complexity of human-nature connectedness.^52^ Theorists look to human experiences as embodied engagements with all things (human and otherwise) and with continually unfolding processes and relations of and with the natural world.^53^ In her exploration of the ‘entanglement’ of matter and meaning through the lens of quantum physics, Karen Barad suggests that humans are part of nature and that practices of knowing are ‘natural processes of engagement with and as part of the world’.^54^ The human experience of the pandemic has emphasised this entanglement. The infection of one human with a novel, serious, and highly infectious disease in one country can have significant and evolving global consequences that play out in relational processes internationally, nationally, locally, and at the level of individual families or households. The experience of a global pandemic is therefore reflective of (and inspires reflection on) such relational entanglements. In the UK, this, we argue, creates space for a renewed ‘relational turn’ both generally and in the NHS (as an organisation built around Bevan’s central idea of communal responsibility) in particular.

As we have explained above, both the NHS and the NHS Constitution build (and are built) on the fundamental importance of relationships, solidarity, and communal responsibility.^55^ Rather than considering each stakeholder as a separately existing, ‘bounded’ individual, the NHS Constitution explicitly takes a relational approach, describing the communities and people the NHS serves—patients, the public, and the staff who work for it—as being ‘bound together’ by the principles and values upon which the NHS as an organisation is built.^56^ The NHS Constitution describes stakeholder responsibilities towards each other *and* to the NHS as an organisation (as to which see further in Section V), and it is on this that we build our argument for looking to the NHS Constitution to underpin and support a relational approach to health and healthcare in the UK. This is especially apposite in the unique circumstances of the post-pandemic new normal.

In the post-pandemic new normal, the importance to communities of individuals’ efforts to stay healthy is likely to be emphasised both by policymakers and by healthcare providers.^57^ Attention will be focused on the social determinants of physical and mental health, with areas of health inequity and disadvantage being a central concern.^58^ A policy framework will be required that is attentive to a relational understanding of individuals’ entanglements within the various communities whose patterns shape, and arguably constrain, their health and their interaction with the NHS. Engagement with concepts, values, and principles that draw attention to the shared interests of individuals and groups will be necessary to reinforce the dimensions of mutuality and relatedness rather than just reflecting the concerns of individuals. Ross Upshur describes these as ‘gluey’ principles.^59^ We argue that the NHS is explicitly underpinned by similar ‘gluey’ ideas, being ‘founded on a common set of principles and values that *bind together* the communities and people it serves’.^60^ It is this that makes the NHS Constitution such an appropriate tool for engaging NHS stakeholders in the ongoing discussions about how best to reorganise the NHS to promote and support an approach that pays attention to relationships and inter-sectionalities.^61^ We argue that the NHS Constitution has to date been under-acknowledged and lacking in practical purpose. Our suggestion is that now is the time to put it to work. The NHS, as an organisation, is currently facing challenges of a magnitude with which it has probably never before had to contend. The pandemic, and the solidarity we suggest, was energised during the first national lockdown, demonstrating the importance of the NHS to the public, as its stakeholders. We do not seek to suggest that enactments of solidarity continue at the level we saw them during the first lockdown.^62^ However, in the context of the cost-of-living crisis that, at the time of writing, is replacing the pandemic in terms of harm being caused to the general public, we consider it equally, if not more, important that public dialogue underpins how to move forward. These are the reasons for which we should harness the potential of the NHS Constitution, in conjunction with a clear and transparent public dialogue, to combat them.

### B. *Solidarity and Care: ‘Gluey’ Relational Values and the NHS Constitution*

Our consideration of the constituting of the NHS in Section II noted Veitch’s suggestion that Bevan’s project was to embed ideas of obligation, solidarity, and care for others into the NHS as a healthcare system.^63^ Our discussion (in Sections IV and V) of the rights and responsibilities the NHS Constitution describes for patients and the public, notes the central importance of *individual* stakeholders being asked to acknowledge some responsibility for the wellbeing of stakeholders *as a group* in their interactions with NHS services.^64^ The NHS Constitution thereby ensures the continuation of Bevan’s project to embed values of solidarity and care into the NHS.

The values of solidarity and care, in the context of the NHS Constitution, are intended to bind NHS stakeholders together with bonds of mutual assistance and shared goals.^65^ They are values that centre the constitutive processes and practices that go on in relationships^66^ and are important not just as ethical theories but as moral *practices*.^67^ In the context of bioethics, (and explicitly in the NHS Constitution^68^) a relational approach ‘strives to place the agency of individuals within a constitutive context of meaning and interdependence’,^69^ where the focus is on the patterns and structures of interrelated activity, choice and the exercise of power in time. Such relational processes and practices are dynamic, powerful, and have the potential to effect change.^70^ They are constituted by, and constituting of, the individuals in whom they are embodied and by whom they are enacted. Thus, solidarity and care, as relational moral practices, are embedded in history and culture. They change (and effect change) over time, and are embodied as ways of living in the natural, material world. The relational values of solidarity and care have been visibly at work in the way patients, the public, and NHS staff have engaged with each other, and with the NHS as an organisation, during the course of the pandemic in the UK.

#### 1. Solidarity

Much has been written on solidarity and there are a variety of understandings of its meaning.^71^ We adopt Jennings’ characterisation of solidarity as an affirmation of others’ moral considerability, and an expression of tangible support for their suffering.^72^ For our purposes, Jasper and Poulson’s suggestion that solidarity can be energised by a ‘moral shock’ is interesting.^73^ They characterise a moral shock as the extreme emotion catalysed by, amongst other things, an unexpected event. In our view, the effects of the COVID-19 pandemic on NHS workers *and* their critically ill patients constituted a moral shock for the public. These unprecedented and unexpected events were experienced by the general public in graphic and upsetting audio-visual packages, widely shared by news and social media. For many, they were personally experienced. This widespread moral shock had various consequences, among them, we suggest, public expressions of solidarity with NHS workers. The participation of the nation in the ‘Clapping for Carers’ that took place every Thursday evening for the duration of the first national lockdown (from 23 March to 28 May 2020) was an early expression of public support for the NHS,^74^ although lacking any meaningful utility in effecting change to healthcare workers’ plight.^75^ More meaningful expressions of solidarity were enacted in the way people set to work making or donating scrubs and other items of essential equipment, the way supermarkets offered priority shopping slots to NHS workers or in the multitude of ‘NHS people offers listed on the NHS website.^76^

We argue that responses like these, enacted across the UK, were reactions to the impossibly difficult physical and moral circumstances in which the NHS (and other) healthcare professionals found themselves. By participating, we suggest that the public and members of the business community expressed solidarity with, and offered their moral support to, healthcare professionals. They also enacted an acceptance of the (reciprocal) responsibility imposed on the general population to abide by the restrictions on their liberty and usual leisure activities as a contribution to minimising the spread of the pandemic, and avoiding added strain on the healthcare effort. Their actions expressed a recognition of the multi-faceted care being offered by healthcare professionals, and offered care in return.

#### 2. Care

The word ‘care’ encompasses a broad range of ideas, theories, and practices.^77^ Here, we use care as a practice complementary to the enactment of solidarity. Where an enactment of solidarity recognises and affirms another’s moral standing, the expression of care, in offering tangible support, pays attention to their needs.^78^ Care theorists describe care as a ‘species activity’; a concrete universal activity that all human beings have in common.^79^ So while care is often enacted within close relationships, it also encompasses a much broader, relational engagement with:

everything we do to maintain, continue and repair our ‘world’ so that we can live in it as well as possible. That world includes our bodies, our selves and our environment, all of which we seek to interweave in a complex, life-sustaining web.^80^

Carol Gilligan, developing her notion of an ethic of care, reflected that moral development is grounded in an individual’s active interaction with both the physical and social world in which they live. She has suggested that an ethic of care ‘guides us in acting carefully in the human world’, paying attention and responding with integrity and respect.^81^ In her later work, Gilligan proposed that healing from trauma requires ‘communilisation of the trauma, being able safely to tell the story to someone who is listening and who can be trusted to retell it truthfully to others in the community’.^82^ During the COVID-19 pandemic, the trauma suffered by healthcare workers engendered a moral shock. The story of their suffering was, in our reading of the national response, heard by the public and retold, nationwide, by the public expressions of solidarity we have highlighted. In enacting solidarity, the public was standing *beside* healthcare workers, recognising and affirming their struggle. In this demonstration of collective appreciation, the public paid attention to their suffering, acknowledging society’s ultimate reliance on healthcare workers and responding with respect.^83^ We suggest that, in making and sharing PPE, for example, the public was doing care work to the extent possible in the constrained circumstances of the pandemic. Some went further, offering direct care to healthcare professionals in whatever way they could.^84^

#### 3. The NHS Constitution: Putting Solidarity and Care to Work

Clearly, over the course of subsequent waves of infection, even though the trauma has continued (and arguably, intensified with time), the moral shock initially catalysed by the first wave of COVID-19 infections subsided, public compliance with infection prevention measures (such as mask-wearing) became less widespread, and public expressions of solidarity and care reduced. Despite public support for protecting the NHS from litigation,^85^ it has been suggested that the NHS will face a large volume of medical negligence claims in response to patients’ treatment during the pandemic, and that other legal claims (such as judicial review of public authority decisions^86^ and claims of human rights abuses) are also to be anticipated.^87^ In the post-pandemic new normal, the NHS will lack both financial and human resources,^88^ and claims against it, if successful, will exacerbate the financial position.

It is crucial that patients and the public appreciate the extent of the challenges the NHS is likely to face, and, specifically, what that means for individual patient care. Our discussion of the patient and public rights and responsibilities described in the NHS Constitution (in Sections IV and V) will touch on this. A re-invigoration of the overt values-based expression of solidarity and care we suggest was enacted and experienced during the first wave of COVID-19, and a discussion about what work these values can do to support the NHS, is timely, and essential. We suggest that the NHS Constitution can support this discussion and help to operationalise an expression of the values of solidarity and care as a public response.

In the UK context, where the public as a whole has a stake in the NHS, this operationalisation would encompass solidarity and *caring* in the context of healthcare very broadly, engaging stakeholders in a conversation on a society-wide level, about how, in the post-pandemic new normal, stakeholders should understand and engage with their rights and responsibilities under the NHS Constitution. We suggest that the value to society of care (and caring) has changed during the national lockdown. We might hold out more hope now than before that, for example, the importance of social care has been elevated, particularly in care homes for the elderly.^89^ Marginalised in the pre-pandemic world, they were at the top of the list for COVID-19 vaccination roll-out.^90^ We might (optimistically) suggest that in the post-pandemic new normal, care in general, and social care in particular, may become more valuable in terms of public policy.^91^ The public affirmation of the importance of care, including social care, their solidarity with the NHS as an organisation and its long-suffering staff, and the visibility in public discourse of the harmful effects of social inequity on society as a whole, suggest that the public may now be receptive to a discussion about how we might, as stakeholders in the NHS, attend more carefully to our responsibilities both to safeguard our own health and to care for others, including those not known to them personally. If we accept the relational values that underpin the NHS, clearly reflected in the NHS Constitution, we can engage with the NHS values (set out in the NHS Constitution) that everyone counts, that everyone’s pain, distress, anxiety, or need matters, that the resources of the NHS are for the benefit of the whole community, and that some people need more help than others.^92^ By accepting a changed, and more onerous, expectation of personal responsibility, we can choose to ‘stand up *with’* the NHS, in a shared commitment to ‘building back better’, where the concept of ‘better’ requires each of us to commit to ‘be our best self’ (and to encourage and support others to do the same) in enjoying the rights *and* accepting the responsibilities the NHS Constitution promotes.

## IV. RIGHTS AND THE NHS CONSTITUTION

### A. *Rights and Values in the NHS Constitution*

The NHS Constitution requires the NHS to respect the human rights of patients and the public, and to promote equality through the services it provides. It preserves the fundamental right of access, free of charge at the point of access, to NHS services, and describes rights to quality (of care and environment), to respect (consent, confidentiality, informed choice, and involvement in healthcare decisions), and a right to complain.^93^ The NHS Constitution affords patients the right to be treated with dignity and respect, and not to be subjected to discrimination. These patient and public rights are situated within a culture where patients are stated to ‘come first in everything the NHS does’.^94^ Integral to the creation of this culture are six ‘core NHS values’ that ‘underpin the NHS’^95^: working together for patients, respect and dignity, commitment to quality of care, compassion, improving lives, and everyone counts.^96^ These are values that ‘patients, public and staff have helped develop’, that ‘inspire passion in the NHS and that should underpin everything it does’.^97^ Both the values and rights set out in the NHS Constitution are intended to be interpreted in the light of seven guiding principles that ‘govern the way that the NHS operates, and defines how [the NHS] seeks to achieve its purpose’^98^:

Principle 1: The NHS provides a comprehensive service available to all;

Principle 2: Access to NHS services is based on clinical need, not an individual's ability to pay;

Principle 3: The NHS aspires to the highest standards of excellence and professionalism;

Principle 4: The patient will be at the heart of everything the NHS does;

Principle 5: The NHS works across organisational boundaries and in partnership with other organisations in the interest of patients, local communities, and the wider population;

Principle 6: The NHS is committed to providing best value for taxpayers' money and the most effective, fair, and sustainable use of finite resources;

Principle 7: The NHS is accountable to the public, communities, and patients that it serves.

These guiding principles, established by the first version of the NHS Constitution, are entrenched by the Health Act 2009: they may not be changed except in accordance with a statutory process.^99^ This process has been used twice,^100^ and in neither case was the centrality of the principles that underpinned Bevan’s vision for the NHS (that it meet the needs of everyone, that it be free at the point of delivery, and that it be based on clinical need, not ability to pay) altered.^101^ Interestingly for our purposes, the changes made to the guiding principles have placed greater emphasis on the ‘centrality of patients managing their own care’,^102^ and the importance of the NHS (as an organisation) taking decisions with patients and local communities.^103^ Thus, the NHS values, and the guiding principles, describe a clear context for the interpretation of the rights and responsibilities of patients and the public. Each patient is important, but the NHS is also concerned with the equitable promotion of health across the wider *community* of patients and the public, and with involving patients and the public (in their capacity as NHS stakeholders) in NHS decision-making.

### B. *Operationalising the Rights and Values in the NHS Constitution*

As we have illustrated above, Bevan’s intention in creating the NHS was to facilitate universal access to the social good of healthcare. The creation of the NHS Constitution recalled and reflected that intention. However, the pandemic has exposed (and exacerbated) significant inequalities in social and economic conditions, including health and access to healthcare.^104^ This suggests that the delivery of NHS services has to date fallen short of the aims, values, and guiding principles in the NHS Constitution. A comprehensive service, available to all, does not seem to have been provided—as the first guiding principle intends. Arguably, now that we are emerging from the pandemic into the post-pandemic ‘new normal,’ there is an opportunity for correction and to consider, explicitly and publicly, how the rights (and responsibilities) described in the NHS Constitution can be put to work to help ‘build back fairer’, as Marmot and others advocate.^105^

The starting point in operationalising the rights in the NHS Constitution is to pay attention to their *meaning*. If patients and the public are to be engaged in a ‘whole stakeholder’ effort to support the NHS in re-imagining what, in the post-pandemic ‘new normal’, a comprehensive service, available to all on the basis of medical need looks like, clarity as to the meaning of patient and public rights and responsibilities will be a key aspect of the discussion. We suggest that a relational approach to understanding and interpreting these rights (and responsibilities) will help provide this clarity, and in so doing, start to address systemic inequities in the healthcare context.

The relational approach we advocate was introduced in Section III. It is underpinned by an acceptance of the fundamental interconnectedness between all humans.^106^ Jennifer Nedelsky, expounding her relational theory, suggests that ‘a path into seeing this interconnectedness is thinking about what harms or benefits each of us,’ stressing that, in the social context, it is essential that we attend to what harms or benefits each of us not as bounded, separate and self-sufficient individuals but *as part of a network of relationships*.^107^ We adopt Nedelsky’s relational approach to reconceiving constitutional rights, developing her analysis of rights and values to provide a framework for our re-view of the NHS Constitution.^108^ We build our argument on the two broad assumptions that underpin Nedelsky’s analysis. First, that rights are best considered in terms of the way they construct relationships—of power, responsibility, trust, and obligation. Secondly, that, in the context of a constitution, rights make most sense viewed as triggers for a dialogue.^109^

#### 1. Rights and Relationships

Our analysis of the meaning of patient and public rights in the NHS Constitution explores those rights which make broad, values-based claims for NHS stakeholders, particularly the rights of access to health services and to quality of care and environment. We adopt Nedelsky’s characterisation of rights and values as linked but distinguishable. Thus, ‘values’ are ethical considerations, ‘used to articulate what a given society sees as essential to humanity or to the good life for its members’ and ‘rights’ are the ways in which an organisation expresses and implements those values in its specific context.^110^ Distinguishing values from rights creates space for a discussion about the nature of any contested rights, the values at stake, what kinds of relationships will foster the organisation’s core values, and how those relationships can support the rights in question.^111^ The values thus become guiding principles for the meaning and implementation of the rights in the context of the relationships in which they are engaged.

The NHS Constitution is underpinned by relational values, as we have seen.^112^ As stakeholders, we are already asked to consider our own interaction with the NHS in the context of the needs of others, including NHS staff, patients, and the wider public.^113^ This is clear both in Bevan’s original ambitions to develop an NHS underpinned by an idea of communal responsibility, and in the intention that NHS Constitution provokes challenge and responsibility among stakeholders for developing and making the best use of NHS services.^114^ Questions about how to make the best use of NHS services will undoubtedly be a significant feature of the post-pandemic new normal. The rights of patients and the public to access services as and when needed will, for some time to come, need to be managed with a view also to reducing the post-COVID-19 waiting lists. If, as we suggest, the public is engaged in a values-based discussion that transparently acknowledges the inevitability of difficult compromises, it might be possible to reduce complaints from individual stakeholders who perceive that their rights to healthcare have been compromised.

The rights the NHS Constitution describes for patients and the public have already been affected by the impact of the pandemic on the NHS. This can readily be illustrated by taking the broad right of access to healthcare services as an example. This right encompasses a range of related rights, including access, free of charge at the point of access, to NHS services.^115^ The NHS Constitution explains to patients and the public that they will not be refused access on unreasonable grounds, and that they will receive care and treatment that is appropriate, meets their needs, and reflects their preferences.^116^ However, what the right of access has meant (and how it has been experienced by patients) in the context of the pandemic has necessarily been quite distinct from its meaning pre-pandemic. Going forward into the post-pandemic new normal, access to services will include the adoption of some pandemic practices, such as ‘virtual’ care via telephone or video-calling, even when the patient prefers to attend a healthcare setting in person. Many patients will be asked to wait much longer than usual for treatment, and it may be that some do not live long enough to receive treatment at all. For those that do receive treatment, the wait might have magnified their needs to the point that curative care is no longer possible, and symptomatic and/or palliative care is all that can be offered. Many will endure considerable suffering for much longer.

Quality of care and environment^117^ is another right that has been experienced differently as a consequence of the pandemic. Infection prevention and control measures aimed at protecting *public* health, such as social distancing, mandatory personal protective equipment, restrictions on visitors and carers accompanying patients into hospital, and requiring people to be tested for SARS-CoV-2 prior to admission (whether as a patient or not), have fundamentally changed the nature of *individual* patient care. Patients’ choices, their preferences, and their wishes and feelings have often (necessarily) been subordinated to the greater good of public health. Public health measures have created a barrier to caring for the individual patient as first concern, as clinical ethics would ordinarily expect, even where the clinical treatment has been equivalent.^118^

Common to both of the above examples is a disruption to the notion that the individual patient has the right, without paying much (if any) attention to the needs of others, to have what they want, in terms of healthcare, and to refuse what they do not want.^119^ Individual patient *autonomy*, often characterised as a broad right to patient *choice*, has been a casualty of the pandemic. A key question for the post-pandemic new normal is how, with constrained resources, continued high demand, and a significant backlog, the NHS is to attend to the healthcare needs of *all* NHS stakeholders. How should the rights of access to NHS services and quality of care be operationalised in the context of the post-pandemic new normal?

An answer to this question can be sketched out if we look back to the discussion space Nedelsky creates by separating constitutional rights and values.^120^ The right of access to NHS services is typically understood as an individual right, underpinned by the key principle of autonomy, understood to be the self-regarding choices individuals make for themselves.^121^ We can, however, re-view this right from the perspective of the core *relational* values that underpin the NHS and inform the NHS Constitution, and a new perspective emerges. By emphasising the importance, in the post-pandemic new normal, of solidarity and care, rather than (individual) autonomy, the patient becomes important not as self-regarding and self-sufficient, but important because of the *relationships* that bind the community of NHS stakeholders. Making visible the symbiotic relationship between values and rights would, we argue, facilitate an appreciation of the necessity for a re-characterisation of stakeholder rights as relational rights, and as an expression of the *communal* responsibility Bevan had in mind. We then need to ask how to go about making that symbiosis visible.

We can start with Nedelsky’s suggestion that the notion of autonomy is also more helpfully situated in the constructive nature of relationships. Nedelsky contends that the structures of relationship in which people are embedded can either prevent the development of autonomy, or allow it to thrive, identifying a lack of information and transparency as preventative factors. We suggest, therefore, that open, collaborative, inclusive, and *informed* engagement of all NHS stakeholders in a discussion about the importance of supportive relationships, and of the ‘gluey’ principles that bind together NHS stakeholders is crucial. This is the first step to developing an enriched understanding of autonomy that acknowledges the importance of stakeholder obligations to each other. For these reasons, transparent dialogue between stakeholders and the NHS (as an organisation) is urgently required.

#### 2. Rights as a Trigger for Dialogue

The notion that rights should trigger dialogue is embedded in the NHS Constitution, and the concepts of dialogue and conversation are fundamental to it.^122^ The NHS Constitution is intended to be dynamic, and to evolve as a result of stakeholder engagement, which is statutorily mandated.^123^ Indeed, while the statutory obligation is that a ‘review’ must be carried out every 10 years,^124^ the NHS Constitution talks about a ‘renewal’, which suggests a more significant change, even a re-promise of the intra-stakeholder rights and responsibilities.^125^ Both words, however, are rooted in an anticipation of, and an allowance for, discussion, development, and change. There is an explicit acceptance that what *ought* to construct the values-based relationships that ‘bind together’ the communities and people served by the NHS will develop over time in response to changing circumstances, and should catalyse a co-produced review (or renewal) of the NHS Constitution.^126^ The unique circumstances of the post-pandemic new normal, and particularly the impact of ‘pandemic values’ on patient and public rights, increase the importance of, and the need for, these relational dialogical practices.

Nedelsky describes dialogue and dialogical processes in the context of constitutional rights as a ‘dialogue of democratic accountability’,^127^ and this idea translates well to the NHS Constitution. Nedelsky suggests that rights are collective choices about the implementation of core values,^128^ and contends that there has been a failure of ‘institutional imagination with regard to the kind of structures that could foster democratic dialogue about the meaning of rights’.^129^ Seeing rights as collective choices makes sense in the context of the fundamental importance of patient and public involvement in reviewing (or renewing) the NHS Constitution.^130^ As to democratic accountability, while this may not be the central aim of the NHS Constitution, the existence of the NHS Constitution, and its insistence on public and patient involvement, represents a structure that *can* foster dialogue about the meaning of the rights it describes. Each review of the NHS Constitution should ensure that attention is paid to what might be described as the ‘organisational ethics’ of the NHS in its delivery of healthcare as a public good.^131^ Raj Mohindra has recently argued that ‘there should be a clear and transparent connection between unambiguous sets of organisational values and the value (in the sense of organisational output and character) that that organisation creates.’^132^ Viewing patient and public rights to NHS services as collective choices about the implementation of NHS values and guiding principles, allows for this clear and transparent connection.

As we move into the post-pandemic ‘new normal’, it is therefore imperative for all NHS stakeholders (staff included) that a new dialogue is started, as soon as possible, to ‘take stock’ of how financial and human resource constraints will impact NHS services. This dialogue should support a transparent re-view of public and patient rights in the ‘reset’ NHS. The principles and values the NHS Constitution proscribes should both set the context and inform the dialogue, emphasising the importance of the relationship between the NHS as an organisation and its stakeholders, and providing an important reminder that ‘the NHS belongs to the people’.^133^ The findings of Marmot and colleagues have shone a light on the damaging interconnectedness between social factors (such as deprivation) and increased vulnerability to severe infection with COVID-19.^134^ Clear approaches to guide the prioritisation of competing rights will therefore be of fundamental importance. Attention must be paid to the causes of inequities more generally, to inequalities within stakeholder communities, and to what the NHS Constitution says to patients and the public about taking responsibility for one another as well as for ourselves.

## V. RESPONSIBILITIES AND THE NHS CONSTITUTION

The NHS Constitution describes a number of patient and public responsibilities in the section that follows the description of their rights, discussed above.^135^ Our focus is on the nature of the responsibilities the NHS Constitution asks of patients and the public (including NHS staff in their capacity as individual stakeholders of the NHS) which, at first sight, seem relatively innocuous. The Handbook describes these responsibilities as, ‘some examples of things that we can all do to make sure that NHS resources are available to everyone who needs them.’^136^ They are to:

- contribute to our own, and our family’s, good health and well-being, and take personal responsibility for it;
- register with a GP practice;
- treat NHS staff and other patients with respect and recognise that violence, or the causing of nuisance or disturbance on NHS premises, could result in prosecution;
- provide accurate information about our health, condition, and status;
- keep appointments, or cancel within reasonable time (because receiving treatment within the maximum waiting times may be compromised unless we do);
- follow the course of treatment to which we have agreed, and talk to our clinician if we find this difficult;
- participate in important public health programmes such as vaccination;
- ensure that those closest to us are aware of our wishes about organ donation; and
- give feedback—both positive and negative—about our experiences and the treatment and care we have received. (Because Feedback will help to improve NHS services for all).^137^

The NHS assumes a ‘model of collaboration between doctors and patients, between the well and the sick, and between patients and patients.’^138^ Patients and the public are asked to accept responsibilities *as* individuals *for* individuals (themselves and other stakeholders) *and for the NHS*. They are asked to accept responsibility for helping the NHS to work efficiently and to ensure that its resources are used responsibly. They are asked to accept responsibility for their actions by, for example, being conscious that if they fail to attend an appointment, that appointment may be ‘wasted’, and someone who might have been seen in their place will have had to wait and may become more unwell as a result. Similarly, they are asked to take part in public health programmes, which are intended to benefit not just the individual stakeholder but society as a whole. It is this focus on *individual* actions to promote collective *public* benefit, already a feature of the NHS Constitution, on which we want to build our suggestions about a re-view of patient and public responsibilities in the post-pandemic new normal.

The NHS Constitution is unusual in asking patients and the public to take responsibility for their health, because, typically, medical ethics does not emphasise patient and public responsibilities.^139^ Heather Draper and Tom Sorell have described medical ethics as one-sided, as it ‘dwells on the ethical obligations of doctors to the exclusion of those of patients’.^140^ Their contention is that, despite often being considered the more vulnerable party in the doctor/patient relationship as a result of the doctor’s learning and social status, patients do (and should) have moral duties and responsibilities in the healthcare context. They situate these responsibilities in the requirements of ‘general ethics’, including the duties of citizens in society and individuals’ responsibilities to others and to themselves.^141^ Some of the wider general responsibilities to which Draper and Sorell appeal are expressly reflected in the NHS Constitution. Examples here include behaving politely to hospital staff, obtaining a repeat prescription in good time, and cancelling a hospital appointment if you can no longer make it.^142^

But to what extent do (or ought) these general responsibilities bind NHS stakeholders to limit, or delay, their use of NHS resources, which we can expect to be scarcer or more in demand, in the post-pandemic new normal and as the grip of the pandemic weakens?^143^ Draper and Sorell have suggested, as Bevan did, that a decent society ought to use what resources it has to ensure that the want of those in need is attended to.^144^ They argue that it is the collective welfare that morally justifies action or inaction, and that as health is an important aspect of welfare, it will often promote public health for individuals to do what they can to stay healthy themselves.^145^ Brazier and Ost, writing in 2013, anticipated such a shift in the private/public balance of rights and responsibilities in the event of a pandemic. They suggested that individual rights would come ‘to be seen as less vital because of the threat posed by those who choose to ignore any responsibility to others’, and that, as a result, ‘public health may more frequently come to outweigh individual liberties’.^146^

In the post-pandemic new normal, this change in emphasis might become visible in various ways, including changed practices or limitations in what is offered. We made reference (in section IV) above to pandemic practices, such as ‘virtual’ care via telephone or video calling, representing a change in stakeholders’ rights of access to NHS services. A relational approach to understanding our responsibilities would encourage accepting virtual care, when requested, even were our preference for a face-to-face consultation, or, conversely, for staff to offer remote consultations where travelling to the hospital would be difficult or expensive and a patient does not need physical examination.^147^ Our responsibilities might also encompass accepting (further) delay to non-urgent treatment for the benefit of those whose needs are more urgent. The COVID-19 vaccination programme is another case in point. Individuals accept vaccinations predominantly for their own benefit, but, in the case of a pandemic, there is also the societal benefit in reducing the risk of further waves of infections.^148^ As this risk reduces, the wider social and resource-based impacts of the pandemic remain to be addressed, as does the question of how each of us, as stakeholders in the (relational) NHS, should understand our responsibility within the context of a society affected unequally by the pandemic.

First on the list of responsibilities that the NHS Constitution asks of us, as stakeholders, is to contribute to our own, and our family’s, good health and wellbeing, and take personal responsibility for it.^149^ Neil Levy has argued, however, that there are grounds for denying that most people are responsible for their own ill health, noting that there is a strong correlation between socioeconomic status and chronic disease, increased risk of morbidity, and early mortality.^150^ This is particularly relevant in the context of the post-pandemic new normal, where the legacy of the pandemic is all around us in unmet health needs, loss of educational opportunities, and financial insecurity.^151^ Levy’s argument, then, is that the challenges of those disproportionately affected by their circumstances should be taken into account in ascribing to them responsibility for their own, and their family members’ health. We can see here echoes of Bevan’s view that financial anxiety is linked to health and recovery from illness, and, accordingly, that healthcare should be a communal responsibility.^152^

In re-viewing the meaning of our responsibilities as NHS stakeholders from a relational perspective, we suggest that the distribution of responsibilities should reflect the same disproportionality. Thus, those who are able to do so should, in the context of health as a public good (and as Levy suggests), be expected to take responsibility for responsibility.^153^ We understand this as an expression of solidarity, an affirmation of others’ moral considerability and an expression of tangible support for their suffering.^154^ What this would require in practice, though, is an understanding (and an acceptance) of the significance of social determinants of health in the broader context of the social fabric, particularly that there is a benefit to society as a whole in ensuring the health of all of its members. This might engage our responsibility as NHS stakeholders in new ways too.

For example, the NHS Constitution states that we have responsibility to participate in public health programmes, such as vaccination. The circumstances of the post-pandemic new normal suggest that, in interpreting the responsibility to participate in such programmes, we should look at the determinants of health more widely, as Marmot and Suleman ask us to do.^155^ Noting the crucial link between food and health, and also between economic circumstances and the ability to make healthy food choices,^156^ such responsibility might include, for example, accepting a greater (fairer?) taxation burden,^157^ contributing to a local food bank, helping out at a soup kitchen for homeless people, or purchasing a ‘Big Issue’ paper from our local vendor. In re-viewing the responsibilities listed in the NHS Constitution in this way, we would be re-interpreting Bevan’s notion of communal responsibility for health in the specific circumstances of the post-pandemic new normal.

Taken seriously, re-viewed through a relational lens, and expanded to fit the circumstances in which the NHS is currently operating, we suggest that patient and public stakeholder responsibilities in the NHS Constitution is therefore considerably more onerous than they initially appear. As discussed above, the significant changes in the way in which the general public has been asked to behave during the pandemic in order to reduce the spread of the virus, and to consider *public* health above *individual* rights and freedom, creates space for a re-view of their *responsibilities* as a collective of individual stakeholders as the NHS emerges from the crisis phase of the pandemic into the post-pandemic new normal.

## VI. CONCLUSION: DRAWING THE THREADS TOGETHER

The NHS has been through a period of significant change over the last decade and the pandemic has changed the game again. As it emerged onto the global stage and surged relentlessly around the world, COVID-19 catalysed an ethical imperative to save as many lives as possible. On the other side of the pandemic, in the reset period and after, a new normal is anticipated, the implications of which will continue to become visible. The pandemic has exposed unacceptable social and health-related inequalities and has energised ethical concerns that are grounded in an acknowledgement of the importance of relational and values-based engagement.^158^ We have argued that an expression of public solidarity was inspired by the morally shocking experiences of NHS workers and NHS stakeholders more widely, and the related distress of the families and friends of those whose lives were not able to be saved. As subsequent waves of infection, and the new variants of the disease, continue to cause illness, hospitalisation, and death, even as people are being vaccinated across the globe, a continued focus on the inequities that create particular vulnerabilities to COVID-19, and how to mitigate them, is essential. The NHS Constitution and the relational, dynamic organisational ethic it describes, offers a workable means of catalysing a conversation about how our rights and responsibilities as stakeholders in the NHS should be re-evaluated through a values-based lens to support an equitable approach to the post-pandemic new normal.

The rights and responsibilities the NHS Constitution describes for patients and the public can be conceptualised as collective choices, co-produced through collaborative dialogue, and informed by the NHS values and its guiding principles. These rights and responsibilities can and should be re-viewed through a relational lens to develop and support ongoing practices of solidarity and care. An approach that accepts the importance of relationality at both an individual and an organisational level, reflects the philosophy underpinning both the NHS as an organisation and the NHS Constitution. It, therefore, offers an appropriate standpoint from which to re-view and revitalise the NHS Constitution, and to reinforce and embed the principles and values that underpin the NHS. Policymakers should support a new engagement between the NHS and the public as its stakeholders.^159^ A transparent, focused, and ongoing dialogue is required to co-construct and embed the rights and responsibilities described in the NHS Constitution as moral practices of solidarity and care. These are essential to support the NHS in the specific context of the post-pandemic new normal.

Sridhar Venkatapuram has recently argued that there has been a singular failure of philosophers and global health policy planners and practitioners to create and engender moral motivation, a will—among those who are able—to create conditions for good health within and across countries.^160^ He quotes John Rawls’ view that one role of political philosophy is to push the limits of what is practically possible in designing political and social institutions.^161^ Pushing the limits, Venkatapuram suggests, means understanding where the world is now and stretching or pulling that towards the best social order that can be imagined; the best world that can be hoped for. We argue that it is timely now for policy planners, practitioners, and the public in the UK to act on Venkatapuram’s suggestion in thinking about the future of the NHS. It is the right time to create and engender moral motivation across stakeholders in the NHS, as we emerge from the pandemic, to stretch and pull towards the best NHS that can be hoped for. Policymakers must push the boundaries of what is practically possible in designing the next chapter for the NHS, and the NHS Constitution can be a tool to support that effort. The Constitution binds together stakeholders in the NHS and anticipates dialogue about change. It is time now to engage stakeholders in a conversation about health services in the context of social conditions more widely construed and about the shape of stakeholders’ rights and responsibilities in whatever context the NHS is operating. Such a conversation, in the context of these challenges, would offer an opportunity to stretch the values of relational solidarity and care enacted by all NHS stakeholders in the first wave of the pandemic into the post-pandemic new normal and beyond.

## Footnotes

1 Michael Anderson and others, LSE–Lancet Commission on the Future of the NHS: Re-Laying the Foundations for an Equitable and Efficient Health and Care Service after COVID-19 (2021) 397 Lancet 1915–78, see eg Recommendation 3C, 1949 <https://doi.org/10.1016/S0140-6736(21)00232-4> accessed 11 August 2021.

2 Department of Health and Social Care, *New TV Advert Urges Public to Stay at Home to Protect the NHS and Save Lives* (Press Release, 10 January 2020) <www.gov.uk/government/news/new-tv-advert-urges-public-to-stay-at-home-to-protect-the-nhs-and-save-lives> accessed 19 April 2021.

3 See Bruce Jennings, ‘Solidarity and Care as Relational Practices’ (2018) 32 Bioethics 553 and, for a general discussion of solidarity, Barbara Prainsack and Alena Buyx, *Solidarity: Reflections on an Emerging Concept in Bioethics* (The Nuffield Council 2011).

4 Department of Health and Social Care, Fourth *report on the effect of the NHS Constitution* (2022) <https://static.primary.prod.gcms.the-infra.com/static/All+Journals/medlaw/Document+Library/oscola-4th-edn-hart-2012.pdf?node=fbf6e4436f73b3b5e77f&version=440095 : a7b7c90bbbe06d7924a5> accessed 8 October 2021.

5 In situating our discussion of the NHS Constitution in the ‘post-pandemic’ new normal, we are describing a normal where (endemic) COVID-19 (and, with any luck, emerging new variants) is (are) contained by effective COVID-19 vaccines, although we note that people are able to choose not to, and some cannot, accept a vaccine.

6 Lucinda Platt and Ross Warwick, *Are Some Ethnic Groups More Vulnerable to COVID-19 than Others?* (The Institute for Fiscal Studies 2020).

7 See, for example, Richard Luscombe, ‘Billionaire David Geffen criticized for tone-deaf self-isolation post’ *The Guardian* (28 March 2020). <www.theguardian.com/us-news/2020/mar/28/billionaire-david-geffen-isolation-coronavirus> accessed 28 September 2021.

8 Mehrunisha Suleman and others, *Unequal Pandemic, Fairer Recovery: The COVID-19 Impact Inquiry Report* (The Health Foundation 2021) <www.health.org.uk/publications/reports/unequal-pandemic-fairer-recovery> accessed 24 May 2022; Michael Marmot and others, *Build Back Fairer: The COVID-19 Marmot Review* (Institute of Health Equity 2020).

9 World Health Organization, ‘Promoting a Fair and Equitable Response to the COVID-19 Pandemic’ <www.who.int/activities/promoting-a-fair-and-equitable-response-to-the-covid19-pandemic> accessed 13 June 2022.

10 Suleman and others (n 8); Marmot and others (n 8).

11 The British Medical Association, ‘July and August 2021 Analysis’ (*Pressure Points in the NHS*, 16 September 2021 <www.bma.org.uk/advice-and-support/nhs-delivery-and-workforce/pressures/pressure-points-in-the-nhs> accessed 8 October 2021).

12 The Patients’ Association, ‘Support for Patients Waiting for Care’ (*Latest News*, 7 October 2021) <www.patients-association.org.uk/News/support-for-patients-wating-for-care> accessed 8 October 2021.

13 Premila Webster and Keith Neal, ‘Covid Reflections—Let Us Talk of Politicians and Professors’ (2021) 43(1) Journal of Public Health 1–2 <doi.org/10.1093/pubmed/fdab075> accessed 19 April 2021.

14 See <https://en.wikipedia.org/wiki/Clap_for_Our_Carers> accessed 19 April 2021.

15 António Guterres, *We Are All in This Together: Human Rights and COVID-19 Response and Recovery* (United Nations 2020) <www.un.org/en/un-coronavirus-communications-team/we-are-all-together-human-rights-and-covid-19-response-and> accessed 19 April 2021.

16 Department of Health and Social Care (n 2).

17 World Health Organization, ‘Older People & COVID-19’ <www.who.int/teams/social-determinants-of-health/demographic-change-and-healthy-ageing/covid-19> accessed 19 April 2021. It is notable that a television drama set in a fictional Liverpool care home in spring 2020, when the coronavirus pandemic swept through the ill-resourced and unprepared care home communities, was prime time viewing in September, 2021. See <www.channel4.com/programmes/help> accessed 23 September 2021.

18 Michael Marmot and others, *Fair Society, Healthy Lives* (The Marmot Review 2010); Michael Marmot and others, *Health Equity in England: The Marmot Review 10 Years On* (The Institute of Health Equity 2020).

19 Marmot and others (n 8). Marmot and colleagues also noted a resurfacing of language of the undeserving poor, perhaps as justification for the regressive changes made to the tax and benefit system. For further discussion of this see Megan Redhead, ‘Malthus’ Enduring Legacy: Poverty, Dependency, and Individual Responsibility in the Nineteenth and Twenty-First Centuries’ (2021) 3 Cambridge Journal of Political Affairs 211.

20 Marmot and others (n 8).

21 For a discussion on the importance of collaboration see Anna Charles, *Integrated Care Systems Explained: Making Sense of Systems, Places and Neighbourhoods* <www.kingsfund.org.uk/publications/integrated-care-systems-explained?utm_source=twitter&utm_medium=social&utm_term=thekingsfund> accessed 11 August 2021.

22 Marmot and others (n 8) see eg Conclusions, p 196.

23 See Jennifer Nedelsky, *Law’s Relations: A Relational Theory of Self, Autonomy and Law* (OUP 2011).

24 A Bevan, *In Place of Fear* (Heinmann 1952).

25 ibid 73.

26 ibid 75.

27 Kenneth Veitch, ‘Obligation and the Changing Nature of Publicly Funded Healthcare’ (2018) 27 MLR 267.

28 Bevan (n 24).

29 ibid 75.

30 ibid 81.

31 ibid 82.

32 ibid.

33 Veitch (n 27).

34 ibid, see s II. Our thanks to an anonymous reviewer of this paper in draft for a helpful comment on this section.

35 Health Act 2009 s 1(1) (a) and see Department of Health, *NHS Constitution—Interactive Version* (DoH 2009) <www.nhs.uk/NHSEngland/aboutnhs/Documents/NHS_Constitution_interactive_9Mar09.pdf> accessed 19 April 2021.

36 Department of Health, *Handbook to the NHS Constitution for England* (DoH 2015) <www.gov.uk/government/publications/supplements-to-the-nhs-constitution-for-england/the-handbook-to-the-nhs-constitution-for-england> accessed 22 September 2021.

37 Health Act 2009 ss 3 and 5, respectively. Both the Constitution and the Handbook have been updated in 2021 (January and February, respectively). Before that, the Constitution was last updated in 2015.

38 ibid s 6. The Fourth report to Parliament was published in January 2022.

39 ibid s 2.

40
*R (Justice for Health) Ltd v Secretary of State for Health* [2016] EWHC 2338 (Admin) at para 88.

41 See discussion in Hannah Gibbs, *The Purpose and Effect of the NHS Constitution* (Landmark Chambers 2017) <www.landmarkchambers.co.uk/wp-content/uploads/2018/06/HG-17-08-30-The-purpose-and-effect-of-the-NHS-constitution-formatted.pdf> accessed 9 April 2021.

42 Department of Health and Social Care, Fourth* Report on the Effect of the NHS Constitution* (Corporate Report 2022).

43 ibid.

44 ibid.

45 ibid.

46 Department of Health and Social Care, *The NHS Constitution for England* (2015) <www.gov.uk/government/publications/the-nhs-constitution-for-england/the-nhs-constitution-for-england> accessed 19 April 2021.

47 ‘The collective principle asserts that…medical treatment and care should be a communal responsibility’. Bevan (n 24) 75.

48 Bevan (n 24) 73.

49 Frank A Chervenak and others, ‘Expanding the Concept of the Professional Integrity of Obstetrics during a Public Health Emergency’ (2020) 48(5) Journal of Perinatal Medicine 435–7.

50 See eg Nedelsky (n 23); Karen Barad, *Meeting the Universe Halfway* (Duke 2007); Mona Livholts and Maria Tamboukou, *Discourse and Narrative Methods: Theoretical Departures, Analytical Strategies and Situated Writings* (Sage 2015); Dian Marie Hosking, ‘Telling Tales of Relations: Appreciating Relational Constructionism’ (2011) 32(1) Organisation Studies 47–65.

51 Nedelsky (n 23) 91–117.

52 See Simon West and others, ‘A relational turn for sustainability science? Relational thinking, leverage points and transformations (2020) 16(1) Ecosystems and People 304.

53 ibid.

54 Barad (n 50) 331–2.

55 ibid 5–8.

56 Department of Health and Social Care (n 46).

57 We note the existence in the USA and elsewhere of Health Maintenance Organisations, which promote initiatives to reduce health expenditure. We are suggesting a values-based approach here, not a system which is motivated by financial gain. For a discussion about HMOs see Roland Petchey, ‘Health Maintenance Organisations: Just What the Doctor Ordered?’ (1987) 16(4) Journal of Social Policy 489–507.

58 Francoise Baylis and others, ‘A Relational Account of Public Health Ethics’ (2008) 1 Public Health Ethics 3, 196.

59 Ross Upshur, ‘Setting the Stage: Population and Public Health Ethics or Public Health Ethics: Ineffable, Ignorable or Essential?’ (Canadian Institutes of Health Research – Institute of Population and Public Health, Population and Public Health Ethics: Cases from Research, Policy, and Practice, University of Toronto Joint Centre for Bioethics, 2012).

60 Department of Health and Social Care (n 46) Introduction (emphasis added).

61 Department of Health and Social Care, *Integration and Innovation: Working Together to Improve Health and Social Care for All* (CP 381, 2019).

62 We note that, in the intervening months, visible enactments of solidarity have waned and that other events, including the ‘partygate’ events the subject of much news reporting at the time of writing, have garnered public attention. This does not negate our argument that a relational, inclusive approach to the significant problem of health inequalities, using the NHS Constitution as a starting point, is a sensible approach.

63 See Veitch (n 27) 271–4.

64 Department of Health and Social Care (n 46) see ‘Patients and the public: your responsibilities’.

65 ibid, see ‘Introduction to the NHS Constitution’.

66 Barad (n 50); Nedelsky (n 23).

67 Jennings (n 3).

68 See Department of Health and Social Care (n 46), ‘Introduction to the NHS Constitution’ and ‘Patients and the public: your responsibilities’.

69 Jennings (n 3) 554.

70 Barad (n 50).

71 See, eg Prainsack and Buyx (n 3).

72 Jennings (n 3).

73 James M Jasper and Jane D Poulsen, ‘Recruiting Strangers and Friends: Moral Shocks and Social Networks in Animal Rights and Anti-Nuclear Protests’ (1995) 42(4) Social Problems 493 and see James M Jasper, ‘The Emotions of Protest: Affective and Reactive Emotions in and around Social Movements’ (1998) 13(3) Sociological Forum 397.

74 See Esther Addley, ‘Clap for Our Carers: The Very unBritish Ritual that United the Nation’ *The Guardian* (28 May 2020) <www.theguardian.com/society/2020/may/28/clap-for-our-carers-the-very-unbritish-ritual-that-united-the-nation> accessed 19 April 2021; Francisco Moura, ‘A United Response to COVID-19: Thank You’ (2021) 102(5) Royal College of Surgeons: The Bulletin 176.

75 Jill Manthorpe and others, ‘Clapping for Carers in the Covid-19 Crisis: Carers' Reflections in a UK Survey’ (2022) 30 Health Soc Care Community 1442–1449 <https://onlinelibrary.wiley.com/doi/epdf/10.1111/hsc.13474> accessed 4 August 2022.

76 NHS England and NHS Improvement, ‘NHS People Offers’ <www.england.nhs.uk/coronavirus/nhs-staff-offers/> accessed 13 June 2022.

77 See, for example, Carol Gilligan, ‘In a Different Voice: Women’s Conception of the Self and of Morality’ (1977) 47(4) Harvard Educational Review 481; Joan Tronto, ‘Beyond Gender Difference to a Theory of Care (1987) 12(4) Signs Journal of Women in Culture and Society 644; Jonathan Herring, *Caring and the Law* (Hart 2013); Nedelsky (n 23); Jennings (n 3); Jean Connolly Carmalt, ‘Human Rights, Care Ethics and Situated Universal Norms’ (2011) 43(2) Antipode 296.

78 Jennings (n 3).

79 See, for example, Eva Feder Kittay, *Love’s Labour: Essays on Women, Equality and Dependency* (Routeledge 1999); Joan Tronto, *Moral Boundaries: A Political Argument for an Ethic of Care* (Routeledge 1994).

80 Joan C Tronto, *Caring Democracy: Markets, Equality and Justice* (New York University Press 2013).

81 Carol Gilligan, ‘Moral Injury and the Ethic of Care: Reframing the Conversation about Differences’ (2014) 45(1) Journal of Social Philosophy 89, 103.

82 ibid 91.

83 Helen Wood and Beverley Skeggs, ‘Clap for Carers? From Care Gratitude to Care Justice’ (2020) 23(4) European Journal of Cultural Studies 641; and see Gilligan (n 81).

84 St George’s University Hospitals NHS Foundation Trust <www.stgeorges.nhs.uk/newsitem/st-georges-icu-nurse-anthea-allen-releases-highly-anticipated-life-death-and-biscuits-diary-of-a-nurse-on-the-covid-19-frontline/> accessed 24 May 2022.

85 Medical Defence Union, ‘Survey Reveals Public Support for NHS to be Shielded from COVID-19 Litigation’ (*MDU Press Release*, 23 June 2020) <www.themdu.com/press-centre/press-releases/survey-reveals-public-support-for-nhs-to-be-shielded-from-covid-litigation> accessed 8 October 2021.

86 This has already started, see, for example, *R (Gardner) v Secretary of State for Health and Social Care* [2022] EWHC 967 (Admin).

87 Sharon Brennan, ‘NHS Told to Expect ‘Huge Number’ of Legal Challenges after Pandemic’ (*HSJ* 2021) <https://www.hsj.co.uk/coronavirus/nhs-told-to-expect-huge-number-of-legal-challenges-after-pandemic/7027448.article> accessed 8 October 2021.

88 Anderson and others (n 1) 1933.

89 See, for example, *Gardner* (n 86); Department of health and Social Care, *Coronavirus (COVID-19) Vaccination of People Working or Deployed in Care Homes: Operational Guidance* (DHSC 2021) <https://assets.publishing.service.gov.uk/government/uploads/system/uploads/attachment_data/file/1010601/coronavirus-covid-19-vaccination-of-people-working-or-deployed-in-care-homes-operational-guidance.pdf> accessed 23 September 202. Note, however, that mandatory vaccination for care home worker raises difficult ethical issues itself.

90 Department for Health and Social Care, *Independent Report: Joint Committee on Vaccination and Immunisation: Advice on Priority Groups for COVID-19 Vaccination* (DHSC 2020) <www.gov.uk/government/publications/priority-groups-for-coronavirus-covid-19-vaccination-advice-from-the-jcvi-30-december-2020/joint-committee-on-vaccination-and-immunisation-advice-on-priority-groups-for-covid-19-vaccination-30-december-2020> accessed 23 September 2021.

91 Although we would not be alone in doing so, see Anderson and others (n 1).

92 Department of Health and Social Care (n 46), see ‘NHS Values’.

93 Department of Health and Social Care (n 46).

94 Department of Health (n 36).

95 ibid.

96 Department of Health and Social Care (n 46).

97 ibid.

98 ibid.

99 Health Act 2009 s 3(7) and s 3(5), respectively.

100 See The National Health Service (Revision of NHS Constitution—Principles) Regulations 2013 and The National Health Service (Revision of NHS Constitution Guiding Principles) Regulations 2015.

101 Bevan (n 24).

102 Department of Health, *Explanatory memorandum to The National Health Service (Revision of NHS*  *Constitution—Principles) Regulations* (Cm 317 2013).

103 Department of Health, *Hard Truths: The Journey to Putting Patients First Volume One of the Government Response to the Mid Staffordshire NHS Foundation Trust Public Inquiry* (Cm 8777-1 2014).

104 Marmot and others (n 8).

105 ibid.

106 See Barad (n 50); Nedelsky (n 23), particularly ch 1; West and others (n 52).

107 Nedelsky (n 23) 232 and see discussion in ch 1.

108 ibid.

109 ibid. Nedelsky’s reconception of rights and constitutionalism considers the protection of rights in a national constitution, and the dialogue to which she refers is a dialogue of democratic accountability.

110 Nedelsky (n 23) 241.

111 ibid.

112 See s 3.

113 See Department of Health and Social Care (n 46), for example.

114 Department of Health and Social Care (n 42).

115 Department of Health and Social Care (n 46) and see Department of Health (n 36) for the fuller description in the Handbook.

116 ibid. See ‘Access to health services: your rights’.

117 ibid, see ‘Quality of care and environment—Your rights,’ and see Department of Health (n 36) for the fuller description in the Handbook.

118 See Lucy Frith and others, ‘Neither ‘Crisis Light’ nor ‘Business as Usual’: Considering the Distinctive Ethical Issues Raised by the Contingency and Reset Phases of a Pandemic’ (2021) 21(8) The American Journal of Bioethics 34.

119 Margaret Brazier and Suzanne Ost, *Bioethics, Medicine and the Criminal*  *Law* (Cambridge University Press 2013).

120 Nedelsky (n 23) 236.

121 Ruth Chadwick and Udo Schüklenk, *This Is Bioethics: An Introduction* (Wiley 2021).

122 Department of Health and Social Care (n 46).

123 Department of Health (n 36) and see Health Act 2009 s 3(2).

124 Health Act 2009 s 3(2).

125 ibid at s 3(2) and the Introduction, respectively.

126 ibid s 3(5). Note, however, that the guiding principles are not generally subject to review.

127 Nedelsky (n 23) 232.

128 ibid.

129 ibid.

130 See Department of Health and Social Care (n 42) and Department of Health and Social Care (n 46) Introduction.

131 For a discussion about organisational ethics in healthcare see Lucy Frith, ‘The NHS and Market Forces in Healthcare: The Need for Organisational Ethics’ (2013) 39 Journal of Medical Ethics 17.

132 Raj Mohindra, ‘‘Value, Values and Valued’: A Tripod for Organisational Ethics’ (2021) 48 Journal of Medical Ethics 3 <https://jme.bmj.com/content/early/2021/03/19/medethics-2020-106837> accessed 25 May 2021.

133 Department of Health and Social Care (n 46), see the opening sentence.

134 Marmot and others (n 8).

135 Department of Health and Social Care (n 46), see ‘Patients and the Public: your responsibilities’.

136 Department of Health (n 36), see ‘Why patient and public responsibilities matter’.

137 Department of Health and Social Care (n 46), see ‘Patients and the Public: your responsibilities’.

138 Margaret Brazier, ‘Do No Harm—Do Patients Have Responsibilities Too?’ (2006) 65(2) Cambridge Law Journal 397, 401.

139 Heather Draper and Tom Sorell, ‘Patients’ Responsibilities in Medical Ethics’ (2002) 16(4) Bioethics 335.

140 ibid 335.

141 ibid 340.

142 ibid; Department of Health and Social Care (n 46) ‘Patients and the public: your responsibilities’.

143 The subject of resource prioritisation is a complex and broad discussion. Our intention is not, in this article, to attempt to engage in this question other than to consider how limited resources and changed access to services might affect the rights and responsibilities of NHS stakeholders as they are described in the NHS Constitution.

144 Draper and Sorell (n 139) 344.

145 ibid 343.

146 Brazier and Ost (n 119).

147 Data from our Reset Ethics research indicates that, for some patients, staff have noted this preference.

148 For information about the potential side effects see NHS Guidance, *Coronavirus (COVID-19) Vaccines Side Effects and Safety* <www.nhs.uk/conditions/coronavirus-covid-19/coronavirus-vaccination/safety-and-side-effects/> accessed 11 August 2021.

149 Department of Health and Social Care (n 46), see ‘Patients and the Public: your responsibilities’.

150 Neil Levy, ‘Taking Responsibility for Responsibility’ in Udo Schüklenk and Peter Singer (eds), *Bioethics: An Anthology* (4th edn, Wiley 2022).

151 Suleman and others (n 8).

152 Bevan (n 24) 75.

153 Levy (n 150).

154 Jennings (n 3).

155 Marmot and others (n 8); Suleman and others (n 8).

156 Suleman and others (n 8).

157 Assuming that the resulting public funds are used to fund appropriate government spending.

158 Marmot and others (n 8).

159 Note that we are not the first to have suggested it. See Anderson and others (n 1) particularly app 2 HL Paper 151, Recommendation 31, 97 <https://publications.parliament.uk/pa/ld201617/ldselect/ldnhssus/151/151.pdf> accessed 11 August 2021.

160 S Venkatapuram, ‘Global health without justice or ethics’ (2021) 43(1) Journal of Public Health 178.

161 John Rawls, *A Theory of Justice* (Harvard University Press 1971).

